# Viral metagenomics reveals two novel anelloviruses in feces of experimental rats

**DOI:** 10.1186/s12985-021-01723-9

**Published:** 2021-12-20

**Authors:** Song-Yi Ning, Ming-Ming Zhou, Jie Yang, Jian Zeng, Jia-Ping Wang

**Affiliations:** 1 Joint Institute of Molecular Etiology Diagnosis, Donghai County People’s Hospital, Jiangsu University, Donghai, 222300 Jiangsu China; 2 School of Medicine, Jiangsu University, Zhenjiang, 212003 Jiangsu China; 3 School of Nursing, Taihu University of Wuxi, Wuxi, 214063 Jiangsu China; 4 Department of Clinical Laboratory, Donghai County People’s Hospital, Donghai, 222300 Jiangsu China

**Keywords:** Experimental rats, Viral metagenomics, Anellovirus, Phylogenetic analysis

## Abstract

**Background:**

Rodents are widely distributed and are the natural reservoirs of a diverse group of zoonotic viruses. Thus, analyzing the viral diversity harbored by rodents could assist efforts to predict and reduce the risk of future emergence of zoonotic viral diseases. Rodents are commonly used in animal testing, particularly mice and rats. Experimental rats are important animal models, and a history of pathogenic infections in these animals will directly affect the animal trial results. The pathogenicity of Anellovirus (AV) remains poorly understood due to the lack of a suitable model cell line or animal to support the viral cycle. This study aimed to discover possible anelloviruses from the virome in feces of experimental rats by viral metagenomic technique.

**Methods:**

Fecal samples were collected from 10 commercial SD rats and pooled into a sample pool and then subjected to libraries construction which was then sequenced on Illumina MiSeq platform. The sequenced reads were analyzed using viral metagenomic analysis pipeline and two novel anelloviruses (AVs) were identified from fecal sample of experimental rats. The prevalence of these two viruses was investigated by conventional PCR.

**Results:**

The complete genomic sequence of these two AVs were determined and fully characterized, with strain name ratane153-zj1 and ratane153-zj2. The circular genomes of ratane153-zj1 and ratane153-zj2 are 2785 nt and 1930 nt in length, respectively, and both include three ORFs. Ratane153-zj1 closely clustered with members within the genus *Wawtorquevirus* and formed a separate branch based on the phylogenetic tree constructed over the amino acid sequence of ORF1 of the two AVs identified in this study and other related AVs. While the complete amino acid sequences of ORF1 of ratane153-zj2 (nt 335 to 1390) had the highest sequence identity with an unclassified AV (GenBank No. ATY37438) from *Chinchilla lanigera*, and they clustered with one AV (GenBank No. QYD02305) belonging to the genus *Etatorquevirus* from *Lynx rufus.* Conventional PCR with two sets of specific primers designed based on the two genomes, respectively, showed that they were detectable at a low frequency in cohorts of experimental rats.

**Conclusion:**

Our study expanded the genome diversity of AVs and provided genetic background information of viruses existed in experimental rats.

## Introduction

Anelloviruses (AV) were first found in the blood of a Japanese patient in 1997 [1]. They have a small covalently closed single-stranded DNA genome. The complete DNA genome ranges from 2 to 3.9 kb in size [2, 3]. AVs are a family of small circular ssDNA viruses with a vast genetic diversity [3, 4]. The virus was first identified as the "TT virus" (TTV), meaning to the patient's initials. It was later called Torque teno virus after the Latin words “torques” (necklace) and “tenuis” (thin) referring to the organization of TTV DNA genome [5]. Since AVs lack their own DNA polymerase, genome replication is entirely dependent on the machinery of the host cell [6], where DNA polymerase forms a double-stranded DNA intermediate during the S (synthesis) process of the cell cycle [7]. As a result, gene replication and ssDNA to dsDNA translation take place in the nucleus [3]. The nucleotide sequence can be used to deduce two key open reading frames (ORFs), ORF1 and ORF2, as well as additional ORFs. These ORFs partly overlap, and their approximate sizes vary greatly among strains [8]. Transfection of TTV DNA into cell cultures, as well as bone marrow cells originating from a TTV positive human, has been used to examine the transcriptional and protein profile of AVs [6]. The study has shown that alternate translational initiation can be used to express at least 5–7 proteins with molecular weights ranging from 12 to 80 kDa [9]. At present, AVs have been identified in several mammals [10], such as pigs [11], dogs [12], masked palm civets [13], rodents [4, 14], bats [15], sea lions [16, 17] and primates [3, 18]. These viruses are highly common, with a mostly stable global spread and a high level of genetic heterogeneity [18]. Lupus, influenza, hematologic conditions, lung diseases, and myopathy are among the major diseases related to AVs [19]. They can be transmitted by sexual routes, blood transfusions, and the fecal–oral pathway [18, 20].

Rodents are widely distributed and are the natural reservoirs of a diverse group of zoonotic viruses [4, 21]. Thus, analyzing the viral diversity harbored by rodents could assist efforts to predict and reduce the risk of future emergence of zoonotic viral diseases. Furthermore, rodents are commonly used in animal testing, particularly mice and rats. Experimental rats are important animal models [22], and a history of pathogenic infections in these animals might directly affect the animal trial results. Viral metagenomics is an unbiased virus-detecting technique that is increasingly applied to non-specifically detect both already known and highly divergent viruses.

Although AVs have been identified in variety of mammal species, the pathogenicity of AV remains poorly understood owing to the lack of a suitable model cell line or animal to support the viral cycle. Here, using the viral metagenomic technique and bioinformatics analysis, we investigated the virome in the feces of experimental rats so as to finding possible AVs, which will be helpful for future establishing animal model for AV infection.

## Materials and methods

### Sample collections and preparations

In this study, samples were collected from ten 10-week-old commercial Sprague Dawley (SD) rats (5 male, 5 female) in Zhenjiang in China in 2019. Ten samples were mixed to construct a virus metagenomic library. Fecal samples were resuspended individually in ten volumes of phosphate-buffered saline (PBS) and vigorously vortexed for 5 min. Supernatant was collected through centrifugation at 15,000 × g for 10 min, followed by filtration of 500 μl of supernatant through a 0.45‐μm filter (Millipore) to remove host cells. The filtrates were incubated with DNase and RNase for 60 min at 37 °C to digest unprotected nucleic acid.

### Viral metagenomic analysis

High-throughput sequencing and bioinformatics analysis was performed according to previous reports [23, 24]. Briefly, the total nucleic acid was then isolated using QiaAmp Mini Viral RNA kit (Qiagen) according to the manufacturer's instructions. Nucleic acid from the 10 fecal samples were pooled into a sample pool which was subjected to library construction using Nextera XT DNA Sample Preparation Kit (Illumina) and sequenced on the NovaSeq Illumina platform with 250 bases paired ends with dual barcodes. For bioinformatics analysis, paired‐end reads of 250 bp were debarcoded. An in‐house analysis pipeline running on a 32‐nodes Linux cluster was used to treat the data. Bioinformatics analysis was performed according to a previous study [25]. Clonal reads were removed and low sequencing quality tails were trimmed using Phred quality score 10 as the threshold. Adaptors were trimmed using the default parameters of VecScreen which is NCBI BLASTn [26] with specialized parameters designed for adapter removal. The cleaned reads were de novo assembled by SOAPdenovo2 version r240 using Kmer size 63 with default settings [27, 28]. The assembled contigs, along with unassembled reads, were compared to an in‐house viral proteome database using BLASTx with default settings except with an E‐value cut‐off of > 10^−5^ [25, 28], where the virus BLASTx database was compiled using NCBI virus reference proteome (ftp://ftp.ncbi.nih.gov/refseq/release/viral/) to which was added viral protein sequences from NCBI nr fasta file (based on annotation taxonomy in Virus Kingdom). Candidate viral hits are then compared to an in-house non-virus non-redundant (NVNR) protein database to remove false-positive viral hits, where the NVNR database was compiled using non-viral protein sequences extracted from NCBI nr fasta file (based on annotation taxonomy excluding Virus Kingdom).

### Phylogenetic analysis

To infer phylogenetic relationships, ORF1 amino acid sequences of reference AV strains belonging to representative genera in *Anelloviridae* family and best matched strains in BLASTp search were downloaded from the NCBI GenBank database. Amino acid sequences were aligned using alignment program implemented in the CLC Genomics Workbench 10.0 with default settings, and the resulting alignment was further optimized using MUSCLE in MEGA v7.0 and MAFFT v7.3.1 employing the E-INS-I algorithm. Bayesian inference trees were then constructed using MrBayes v3.2 [29], where we set “prset aamodelpr = mixed” for the phylogenetic analysis, which allows the program to utilize the 10 built-in amino acid models. The Markov chain was run for a maximum of 1 million generations, in which every 50 generations were sampled and the first 25% of Markov chain Monte Carlo (mcmc) samples were discarded as burn-in. Resulted phylogenetic tree was viewed and edited in FigTree v1.4.2 software.

### PCR screening

After bioinformatic analysis, two putative novel AVs were discovered in the library and named ratane153-zj1 and ratane153-zj2, respectively. To investigate whether the 2 novel AVs were prevalent in experimental rats, two additional batches of samples were collected from the same sampling site as the 1^st^ time of sampling. The 2^nd^ time of feces sampling from other 13 SD (7 male and 6 female) rats was performed one month after the original sampling and the 3^rd^ time of sampling from the other 15 SD rats (7 male and 8 female) rats was performed one month after the 2^nd^ time of feces sampling. Besides, the original 10 samples including in the library construction were also checked. PCR screenings of the two AVs were performed with primers designed based on the genome sequences of ratane153-zj1 and ratane153-zj2. PCR primer sequence and parameter for each specific virus are showed in Table 1. The specific DNA bands were T-A cloned and sequenced by Sanger method. Standard precautions were used for all procedures to prevent the cross-sample contamination.

**Table 1:** Nested PCR primers designed based on ORF1 for screening positive samples

Virus	Primer	Sequence	Amplified product (bp)	Tm (℃)
ratane153-zj1	WF	CTTCTGGTGGAGCACAGAGG	536	48
	WR	AATACCAAGCAGCAGGCCAT		
	NF	ATCATTCACAGAAGCGGCCA	312	53
	NR	AGCAAGGTCTCGTATTCCGC		
ratane153-zj2	WF	GTTCGTGACCCAGACAACCT	492	48
	WR	AGATTCCTGCCTCCCCATCT		
	NF	AGCAGAGATAGGGTAGGGCC	277	55
	NR	GCTCGGCTTCTGACAGAGTA		

## Results

**Fig. 1:**
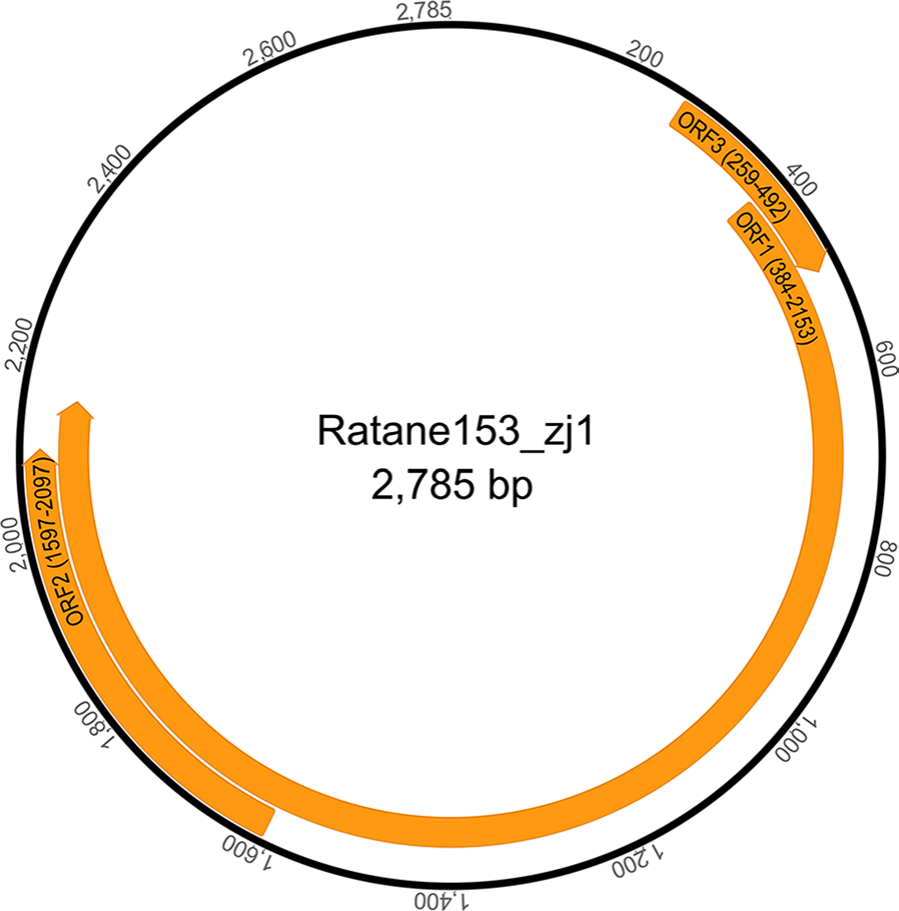
Genomic organization of the novel anellovirus ratane153-zj1 detected in fecal sample of experimental rats

**Fig. 2:**
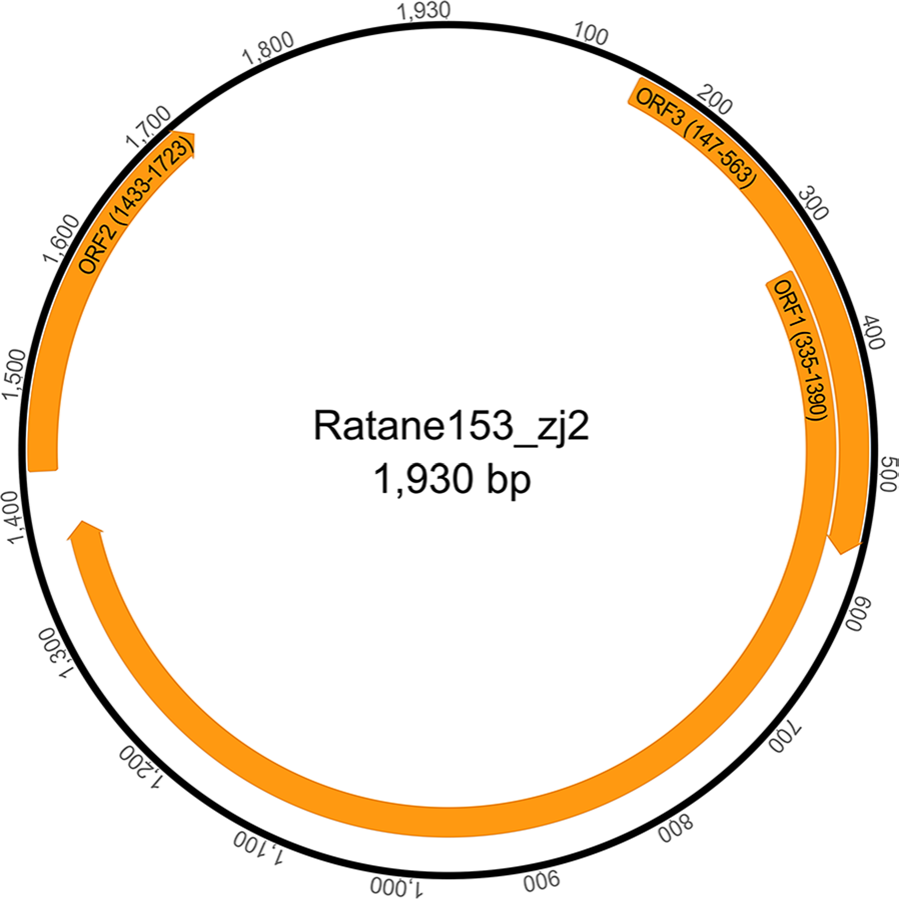
Genomic organization of the novel anellovirus ratane153-zj2 detected in fecal sample of experimental rats

The fecal sample pool from 10 experimental rats generated a total of 4,460,218 unique sequence reads. Sequence reads were de novo assembled and compared to the GenBank nonredundant protein database using BLASTx. Results indicated that two contigs showed significant similarity to known AVs, which showed overlapping regions at the start and end of the contigs, confirming their circular genomes. The two complete viral genomes were named ratane153-zj1 (Fig. 1) and ratane153-zj2 (Fig. 2), respectively. The circular genomes of ratane153-zj1 and ratane153-zj2 are 2785 nt and 1930 nt long, with a G + C content of 50% and 46%, respectively. The ORF1, the largest ORF in these two AVs, encodes a 589 amino acid long putative capsid protein in ratane153-zj1 and 351 amino acids in ratane153-zj2, respectively.

**Fig. 3:**
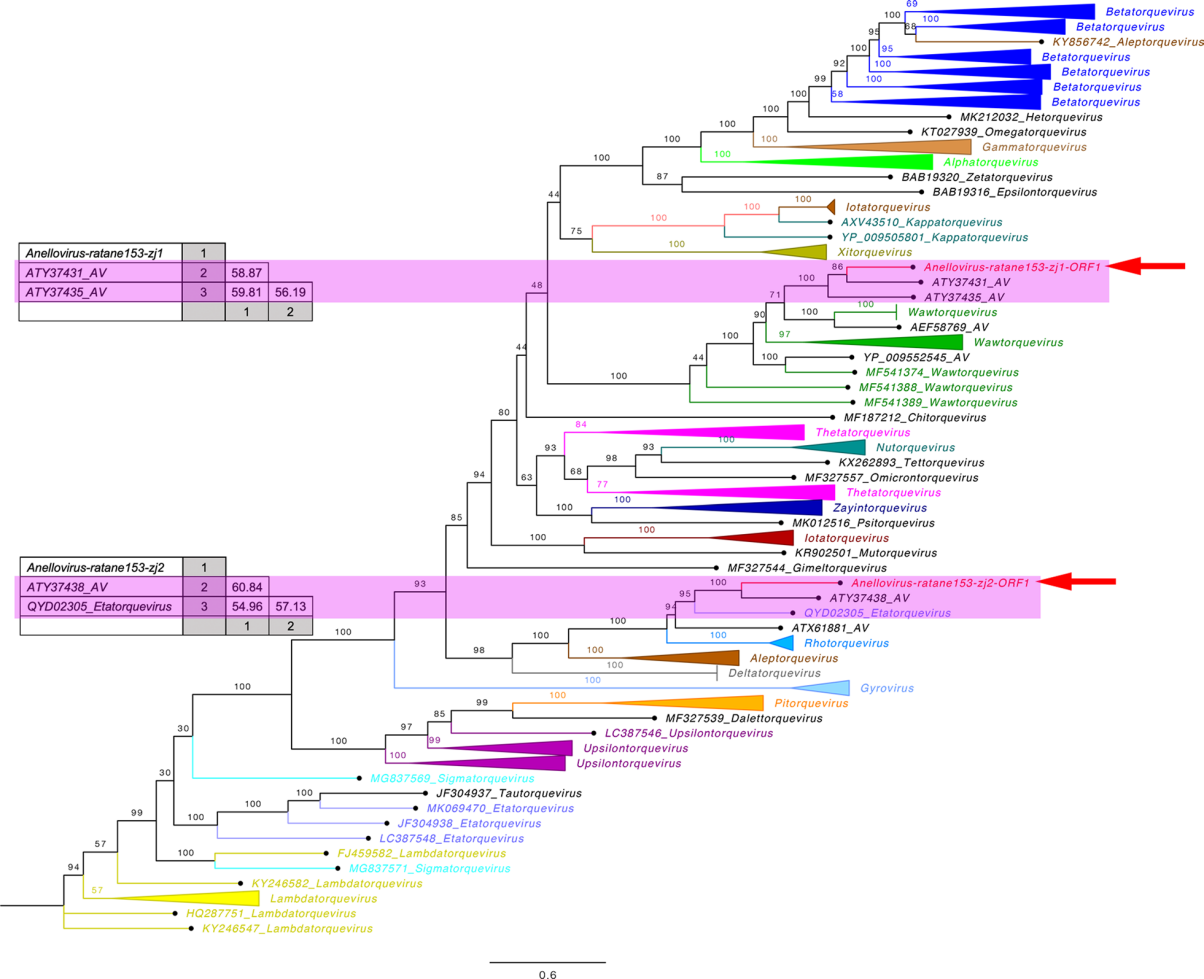
Phylogenetic tree established based on the amino acid sequences of the ORF1 region of ratane153-zj1, ratane153-zj2 and representative members of the family *Anelloviridae*. Red arrows indicate the two novel AVs identified in this study. Pink rectangles represent the pairwise sequence identity between the two novel AVs and the other related virus strains based on nucleotide sequences of ORF1

To determine the relationship of these two novel viruses to other AVs, a phylogram was created based on the amino acid sequences encoded by ORF1. The phylogenetic analysis indicated that the two AVs identified in fecal samples of experimental rats were clustered into two distinct groups of AVs which were discovered from different wild species in the order Rodentia [14]. Besides, pairwise sequence comparisons based on the complete nucleotide sequences of ORF1 (nucleotides [nt] 384 to 2153, Fig. 1) showed that ratane153-zj1 shared the highest sequence identity of 59.81% to an unclassified AV (GenBank No. ATY37435) from wild *Neodon leucuru* (Fig. 3). Ratane153-zj1 closely clustered with members within the genus *Wawtorquevirus* and formed a separate branch (Fig. 3). While the complete nucleotide sequences of ORF1 of ratane153-zj2 (nt 335 to 1390, Fig. 2) had the highest sequence identity of 60.84% with an unclassified AV (GenBank No. ATY37438) from wild *Chinchilla lanigera*, and they clustered with one AV (GenBank No. QYD02305) belonging to the genus *Etatorquevirus* from *Lynx rufus.*

To investigate the prevalence of these 2 novel AVs in cohorts of experimental rats, two additional batches of fecal samples were collected with sample sizes of 13 and 15, respectively, from the same sampling site. A total of 38 samples (including 10 samples of the original samples) were subjected to PCR screening using primers showed in Table 1. For ratane153-zj1, 2 out of 10 samples from the original samples were positive while the other 28 samples were all negative. For ratane153-zj2, there was only one positive sample from the original samples and 2 out of the 28 samples collected after library construction. The gel electrophoresis bands were T-A cloned and sequenced by Sanger method. Sequencing results confirmed the existence of the AVs in these samples.

### Nucleotide sequence accession numbers

The raw sequence reads from the metagenomic library were deposited in the Short Read Archive of GenBank database with accession no. SRX10073590. The 2 genome sequences characterized in detail in this study were submitted to GenBank with the accession nos. MW644973 and MW644974, respectively.

## Discussion

Virome investigation based on sampling from patients with immunosuppression and antiviral therapy revealed that the human plasma virome was composed of seven distinct viral families where the dominant virus family, the *Anelloviridae*, accounted for 68% of the total virus population and 97% of the AVs belonged to the *Alphatorquevirus* genus [30]. Here, we report two novel AVs, ratane153-zj1 and ratane153-zj2, that were discovered in feces of rats. Phylogenetic analysis showed that 153-zj1 clustered within a group that included the one AV from wild *Neodon leucuru,* and ratane153-zj2 clustered within a group that included the one AV from wild *Chinchilla lanigera.* AVs are subgrouped into Torque teno viruses (TTV), Torque teno mini virus (TTMV), Torque teno midi virus (TTMDV) and small anellovirus (SAV) in human [31]. Given their sequence divergence, two novel AVs identified in this study can be assigned as new rat AVs species. The latest published report (2020 Release) from the International Committee on Taxonomy of Viruses (ICTV), lists 155 species of AVs in the family *Anelloviridae* [32]. Based on the taxonomic classification criteria of ICTV (i.e. cut-off values for sequence divergence of entire ORF1: genera > 56%, species > 35%) [33], we deduce that ratane153-zj1 belongs to the genus *Wawtorquevirus* and ratane153-zj2 is a member within the genus *Etatorquevirus*.

AVs have been found in many vertebrate hosts including primates [3, 8, 24]. They have also been found in invertebrate mosquito, most likely because of mosquito blood feeding from vertebrate hosts [34, 35]. In addition, the Chicken anemia virus is a pathogen of chickens with a global spread that belongs to the *Anelloviridae* family [36]. Rodents act as important natural hosts for the transmission of a wide variety of viruses in the wild [21]. AVs were found in most rodent animals in China and UK, especially in the genera *Rattus*, *Mus*, *Niviventer*, and *Apodemus*, which had a higher AV-positive rate at the pool level [4, 14]. In this study, we describe the application of the high-throughput sequencing to examine the frequency of AVs in rats. Screening results showed these two AVs were detectable at a low frequency in these 38 feces of experimental rats. These results also provide a firm base for detecting and monitoring possible rodent-origin AVs.

The pathogenicity of AVs remains poorly understood because of the lack of a suitable model cell line or animal to support the viral cycle [14]. Large experimental animals such as pigs are costly to treat and maintain in terms of reagents and resources [11]. This study results indicated that experimental rat carries the rodent AVs, suggesting that laboratory rat be infected by these two novel AVs, which could be used in studying the viral cycle and pathogenicity.

## Conclusion

Using viral metagenomics, two novel AVs were identified from fecal sample of experimental rats. The complete genomes sequence of these two AVs was determined and fully characterized, with strain name ratane153-zj1 and ratane153-zj2. Ratane153-zj1 closely clustered with members within the genus *Wawtorquevirus* and formed a separate branch based on the phylogenetic tree while ratane153-zj2 belongs to the genus *Etatorquevirus*. Conventional PCR with two sets of specific primers designed based the two genomes, respectively, showed that they were detectable at a low frequency in cohorts of experimental rats. Our study expanded the genome diversity of AVs and provided genetic background information of viruses existed in experimental rats, also provide a meaningful basis for the identification and tracking of potential rodent-origin AVs.

## Data Availability

The raw sequence reads from the metagenomic library were deposited in the Short Read Archive of GenBank database with accession no. SRX10073590. The 2 genome sequences characterized in detail in this study were submitted to GenBank with the accession nos. MW644973 and MW644974, respectively.

## Abbreviations

BLAST: Basic local alignment search tool
AV: Anelloviruses
NCBI: National Center for Biotechnology Information
ORF: Open reading frame
ICTV: International Committee on Taxonomy of Viruses
TTV: Torque teno viruses
TTMV: TTV-like mini virus
TTMV: Torque teno mini virus
TTMDV: Torque teno midi virus
SAV: Small anellovirus
